# Microbiome, Immunosenescence, and Chronic Kidney Disease

**DOI:** 10.3389/fmed.2021.661203

**Published:** 2021-03-19

**Authors:** Elisavet Stavropoulou, Konstantia Kantartzi, Christina Tsigalou, Konstantina Aftzoglou, Chrysa Voidarou, Theocharis Konstantinidis, Mariana Carmen Chifiriuc, Elias Thodis, Eugenia Bezirtzoglou

**Affiliations:** ^1^CHUV (Centre Hospitalier Universitaire Vaudois), Rue du Bugnon, Lausanne, Switzerland; ^2^Department of Infectious Diseases, Central Institute, Valais Hospital, Sion, Switzerland; ^3^Nephrology Clinic, Department of Medicine, Democritus University of Thrace, Alexandroupoli, Greece; ^4^Laboratory of Microbiology, Department of Medicine, Democritus University of Thrace, Alexandroupoli, Greece; ^5^Medical School, Comenius University, Bratislava, Slovakia; ^6^Department of Public Health P.U., Arta, Greece; ^7^Department of Medicine, Democritus University of Thrace, Alexandroupoli, Greece; ^8^Laboratory of Microbiology, Faculty of Biology, The Research Institute of the University of Bucharest (ICUB), University of Bucharest, Bucharest, Romania; ^9^Laboratory of Hygiene and Environmental Protection, Department of Medicine, Democritus University of Thrace, Alexandroupoli, Greece

**Keywords:** gut, kidney, gut-kidney axis, microbiome, ageing, immunosenescence, chronic kidney disease, microbiota

## Abstract

The gut microbiome is known as an important predictive tool for perceiving characteristic shifts in disease states. Multiple renal diseases and pathologies seem to be associated with gut dysbiosis which directly affects host homeostasis. The gastrointestinal-kidney dialogue confers interesting information about the pathogenesis of multiple kidney diseases. Moreover, aging is followed by specific shifts in the human microbiome, and gradual elimination of physiological functions predisposes the microbiome to inflammaging, sarcopenia, and disease. Aging is characterized by a microbiota with an abundance of disease-associated pathobionts. Multiple factors such as the immune system, environment, medication, diet, and genetic endowment are involved in determining the age of the microbiome in health and disease. Our present review promotes recently acquired knowledge and is expected to inspire researchers to advance studies and investigations on the involved pathways of the gut microbiota and kidney axis.

## Introducing the Aging Microbiome

The human newborn is devoid of bacteria at birth (1). Bacteria colonizing the sterile newborn either come from the hospital environment and staff as in the case of caesarian section or from normal maternal vaginal microflora (1). The establishment and progression of the human microflora is attributed to the influence of multiple epigenetic mechanisms (2). Personal habits and behavior, stress, hormones, antibiotics, vaccination, and infections (1) seem to be involved. However, nutrition remains the ultimate factor that can sway newborn development processes regulating epigenetic mechanisms during pregnancy and early life (1, 2). The importance of food intake variations is stated by a plethora of publications (1–4). Studies comparing children in rural Africa and Europe reported important variations in microbial populations due to eating habits (5). African children were colonized by more bacteria belonging to *Actinobacteria* phylum and *Bacteroidetes* than *Firmicutes* compared to European children group who carried more *Firmicutes and Proteobacteria*.

The importance of early life colonization is understood (1). The presence of beneficial bacteria such as *Lactobacilli* and *Bifidobacteria* protect against disease (6). The “Hygiene Hypothesis” was advanced to explain atopic disorders after immune dysregulation (7). Human microbiota evolve in parallel with the immune system supporting a bidirectional relationship resulting in normal immune development (8).

Nowadays, the term “microflora” is used less frequently in favor of the term “microbiota” as microbial genomes are also involved. The term was first used by the Nobel Laureate Joshua Lederberg (9).

Bacterial communities are involved in complex inter-communication and network models of unique microbiomes. In this vein, characterization of the different microbial communities in health and disease status was achieved due to new technological involvements and particularly 16S rRNA sequencing. This methodology permits the identification of complex microbial populations in the human body (10). Additionally, metagenomics Whole Genome Shotgun (WGS) sequencing has allowed for the identification of involved functions in relation to our microbiome (10, 11). It seems to be less crucial to confirm “who is there” than “what are they doing.” The *Human Microbiome Project* (USA) (12) as well as *the metaHIT Consortium* (Europe) (13) have shed light on the characterization of major healthy human sites in order to compare them with shifts occurring in disease states (14).

In developed countries, during the last century, improvements in healthcare have led to a population of higher age and life expectancy has risen (15). With the recognition of an aging population (16), geriatric research has gained the interest of multiple society sectors including topics such as social, work, and economic impact and nutrition and health issues.

It is known that frail and elderly people encounter more infections than younger people (17). Infections in elderly subjects are often complicated due to multi-morbidity (17), hormonal shifting, increased production of pro-inflammatory cytokines and chemokines, and abnormalities of the telomeres which finally could cause a dysfunction of the immune system called immunosenescence and malnutrition.

The impact of aging upon the intestinal microbiota is associated with a decrease in the anaerobic population (18, 19), specifically the *Bifidobacterial* population (20, 21), while an increase in E*nterobacterial* population has been reported (19, 21).

Age-related sequential changes were reported in the human microbiota (22) by 16SrRNA methodologies. *Actinobacteria* phylum (mainly *Bifidobacteria*) was decreased with age and after weaning (22), while *Firmicutes* (mainly *Clostridium* cluster XIVa and *Faecalibacterium* 57 *prausnitzii*) were more frequent in older children but at lower levels (23). Finally, *Bacteroidetes* and *Proteobacteria* were found in human recipients over 70 years old (22). Taking it one step further, in analysis focusing on bacterial co-abundance groups (CAGs) as defined by Kendall, correlations between genera showed that several transition types of microbiota were enriched in the adult population (22). Relative abundance of genera was registered in elderly-associated CAGs compared to infant- and adult-associated CAGs (22). Linkage clustering based on the abundance of genera indicated five age clusters with median ages 3, 33, 42, 77, and 94 years old (22). However, when clustering was based on the proportion of transporters evaluated by phylogenetic analysis of the bacterial communities by reconstruction of unobserved states (PICRUSt), the human recipients were classified into two age groups; the adult-enriched and the infant/elderly-enriched clusters (22).

## Immunological Pathways in Kidney Disease

It is known that the intestine possesses dual functions, firstly a role in nutrient absorption and also a function in the synthesis of substances such as amino acids, vitamins, and short chain fatty acids (SCFAs) (24). SCFAs exert beneficial effects, confer energy to epithelial cells, and engage in a potent role in the immunomodulation and barrier effect against pathogenic invaders (24). Particularly, they hold two basic signaling functions; the activation of G-protein-coupled receptors (GPCRs) and the inhibition of histone deacetylases (HDACs) (25). GPCRs are receptors of SCFAs which participate in metabolism, inflammation, and disease processes (25). Still, SCFAs are activated in the free fatty acid receptor-2 and−3 (FFAR2 and FFAR3) found in numerous human body sites (26). Additionally, SCFAs upset the physiology of the intestinal epithelial cells by inhibiting histone deacetylases (HDACs) resulting in chromatin remodeling and changes in transcription processes (27). Finally, HDACs seem to possess an anti-proliferative and anti-inflammatory action either *in vitro* or *in vivo* in developed models of inflammation (27).

In this vein, the intestinal microbiota *via* the intestinal barrier seem to adjust homeostasis and functions of both innate and adaptive immunity locally and systemically (28). However, when the intestinal barrier is breached, a situation called “leaky gut,” the gut bacteria and their toxins are able to infiltrate the intestinal mucosa and then through the blood stream circulate to different tissues and organs (1, 29). Moreover, activated immune cells penetrate the kidney and generate pro-and anti-inflammatory reactions and regulatory signals in order to induce a neutrophils response (30). Neutrophils together with macrophages are induced as part of the first line response in innate immunity against pathogens (31) and kidney disease (32).

Impairment of the macrophages' phagocytic ability has a negative effect on kidney function leading to chronic inflammation (31). Chronic systemic inflammation can be appraised using the neutrophil-to-lymphocyte ratio (NLR) which is associated with the risk of ESRD with stage 4 CKD. NLR could be a prognostic marker for cardiovascular risk and mortality in patients with CKD 3-5 and hemodialysis-peritoneal dialysis patients, respectively (33, 34).

To this end, the role of pattern recognition receptors (PRRs), and especially TLRs (toll like receptors) which are membrane glycoproteins, during inflammation processes is stated (31). TLRs are found in renal cells and activate mitogen-activated protein kinases, nuclear factor-κB, and activator protein-1 toward a pro-inflammatory status (35, 36). The importance of a dialogue between the acquired immune system and the innate system is understood (37, 38) through the production of cytokines.

Renal tubular epithelial cells participate in immunity processes by producing chemokines, cytokines, and antimicrobial substances (32). In their turn, cytokines participate in the immunological response by promoting the synthesis of acute phase proteins and tissue proteolysis and lipolysis. Moreover, they interact with T lymphocytes to generate the acquired immune response (39). A cell-mediated response to the antigen will take place and T lymphocytes will similarly produce cytokines in order to regulate the activity of immunocompetent cells and induce antibody production (40). Injured renal tubular epithelial cells dedifferentiate to achieve refit and thus they incite inflammation by recruiting myofibroblasts. In this way, tubular epithelial cell loss stimulates residual renal hypertrophy. Thus, the hypertrophied nephron is unable to cope with the increase in tubular transport as it overwhelms its energy-generating capacity, and anaerobic metabolism, acidosis, and hypoxia occurs (41, 42).

Renal tubular epithelial cells present a crucial role in inflammation, positively or negatively regulating T cell responses in an alternative way, as they express co-stimulators of T cells (ICOS-L) and B7-H1 molecules (43). Yet, macrophages, dendritic cells (DCs), and T regulatory cells (Tregs) induce an adaptive immune response and DC activation promotes the production of proinflammatory cytokines such as IL-12 and IL-6 (30). Clearly, DCs trigger the differentiation of naïve CD4^+^ T cells into regulatory T (Treg) cells and the maturation of B cells into IgA-secreting ones (30, 44). The role of Treg cells in renal disease seems to be crucial in protecting against inflammation processes and amplifying homeostasis by boosting microbiota (45). In support, T helper 17 (Th17) cells are activated inducing the production of pro-inflammatory interleukin-17 (IL-17) (46). To this end, nuclear factor-κB (NF-κB) is released by the renal tubular epithelial cells regulating pro-inflammatory response (47).

Furthermore, innate lymphoid cells (ILCs) tamper with pro-inflammatory cytokines IL-1β, IL-12, IL-23, IL-22, and IFNγ production (44, 48). It was also found that the aryl hydrocarbon receptor of IL-22 in innate lymphoid cell response (ILC3) suppresses inflammatory Th17 cell responses and regulates Treg-mediated gut homeostasis (49). So then, the suppression of Th17 cells in the intestine confers positively to the translocation and activation in the kidney (44). Gut expressed Th cells can be activated in the kidney through the CCL20/CCR6 axis (50).

**Figure 1:**
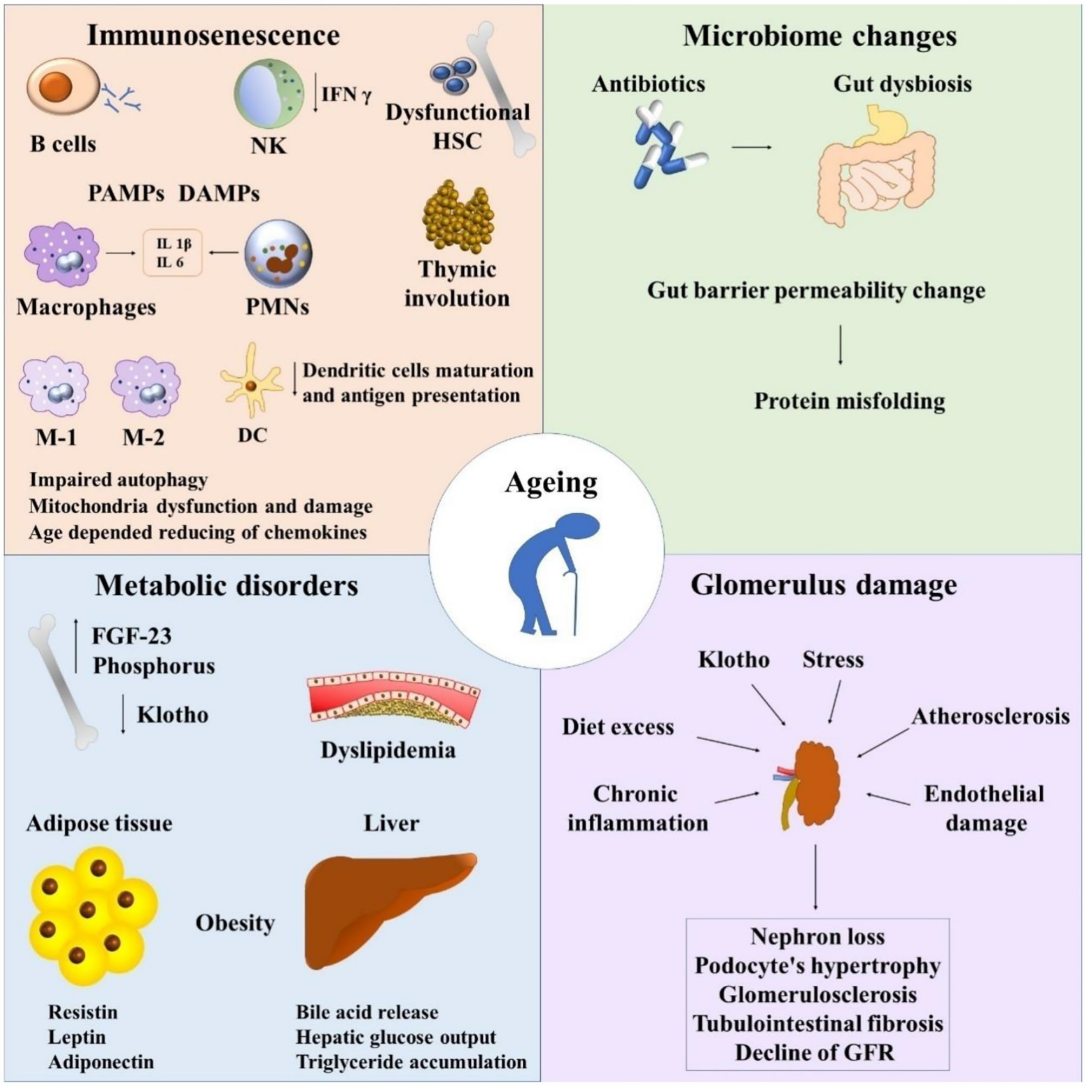
Immunosenescence and chronic kidney disease. NK, natural killers; HSC, hematopoietic stem cells; PAMPs, pathogen-associated molecular pattern molecules; DAMPs, damage-associated molecular pattern molecules; PMNs, polymorphonuclear leukocytes; FGF-23, fibroblast growth factor 23; GFR, glomerular filtration rate.

The intestinal microbiota cooperate by means of microbial associated molecular patterns (MAMPs) or SCFAs as previously discussed to temper inflammation in the kidney (51). It is of note that by the aid of RT-PCR, four receptors (GPR41, GPR43, Olfr78, and GPR109a) expressed in the kidney are linked to particular pathologies (52) (Figure 1).

Without any doubt, important physiological changes occur in the kidney as a result of immunoactivation. Immune cells and inflammatory proteins contribute to the pathogenesis of kidney diseases (53). Finally, it is worth noting the importance of the dialogue between the kidney and gut, the so-called gut-kidney axis in health and disease (54).

Actually, in spite of the technological advancements in peritoneal dialysis (PD) and hemodialysis (HD) procedures, the mortality in ESRD remains high (55) as cardiovascular disease and infections occurred in these patients. It seems that both complications are associated with immunological shifts in ESRD such as uremia (55). Uremia is characterized by immune dysfunction and immunosuppression leading to multiple infections. The accumulation of pro-inflammatory cytokines takes place as a result of dropped renal elimination capacity, oxidative stress, and the accumulation of uremic toxins. Moreover, immunoactivation results in inflammation and cardiovascular disease. Immune dysfunction in uremia is linked to both innate and adaptive immunity (55). Yet, adaptive immunity is altered in ESRD patients. It seems to be caused by uremia *per se* and chronic renal failure. T cell proliferation is mitigated in an uremic environment. T helper lymphocytes (Th) have an impact on the immune response. Th1 cells activate macrophages, while Th2 cells promote humoral immunity (56). Interestingly, the maturation of Th cells in hemodialysis patients (HD) does occur, these subjects showed increased Th1 concentrations and an increased Th1/Th2 ratio (57). Studies state that ratio increase in HD is associated with the elevated production of IL-12 which effects T lymphocytes. This leads to an increase in IFN-γ and a decrease in IL-4, promoting their differentiation in Th1 cells (55). Yet, B cell lymphopenia is apparent due to apoptosis, despite the production of IgM and IgA in normal levels in dialysis patients (58). Following initiation of renal replacement therapy in HD or peritoneal dialysis (CAPD) subjects, the immunological status of patients was appraised (59). The percentage of CD4+CD28 null and CD8+CD28 null cells was found increased in ESRD patients. Therefore, CD4+CD28 null cells correlated with CRP and serum albumin levels while important differences in items of CD4+CD28 null and CD8+CD28 null cells were found in patients with cardiovascular disease. Shifts in the population of CD4+CD28 null cells was found following 6 months of dialysis. However, these changes showed significant differences between HD and CAPD patients (59), T cells subtypes are affected by CKD and a chronic inflammation disease is installed. This turmoil is enhanced in HD patients but alleviated in CAPD patients (59).

The intestinal microbiome of HD patients showed an increase in *Proteobacteria, Actinobacteria*, and *Firmicutes* with preponderance of the *subphylum Clostridia*, while a decrease in the *taxa Firmicutes* and *Actinobacteria* is found in CAPD patients (60). It is known that there is an interplay between the kidney and gut, called the gut-kidney axis (54, 61). Renal transplantation incites changes in the gut microbiota (62). Yet, hormones, environment, genetics, epigenetics, and pharmacogenetics seem to impact kidney allograft receivers (62).

Gut microbiota could incite antigen-presenting cells (APCs) and initiate immune response and alloimmune reactivity, as is the case in allogeneic bone marrow transplantation (HSCT) (63). However, when allograft recipients are submitted to gut decontamination, acute graft vs. host disease declines (64). A considerable shift in microbiota was found 1 month after transplantation. It is of note that patients hosting *Faecalibacterium prausnitzii* in their microbiota need higher tacrolimus therapeutic doses (65).

Researchers found that gut-associated lymphoid tissue (GALT) plays a key role in the evolvement of immunoglobulin A (IgA) nephropathy (IgAN) (66).

Changes in gut microbiota and dysbiosis seem to be critical for immunoglobulin A nephropathy (IgAN) (54). In IgAN patients, an abundance of *Fusobacteria* is observed, while *Synergistetes* were decreased (67). Genome studies showed that IgAN and inflammatory bowel diseases are linked to the same loci (66). This observation involves a different clinical approach including a treatment option that focuses on subclinical intestinal inflammation or microbiota shifting (68).

Dysbiosis of the gut microbiota was also related to patients with idiopathic membranous nephrotic syndrome (INS) (69). *Fusobacteria, Proteobacteria*, and *Parabacteroides* are increased in INS patients, while *Firmicutes* dropped (69). At the genus level, ***Providencia*** and ***Myroides*** were found more frequently in INS patients (69). Yet, propionate acid and butyric acid are found in low concentrations in INS patients (69).

## Aging and Senescence: Two Facets in the Context of Immunity

During aging, physiological and pathological changes emerge in contrast to senescence where mainly non-pathological changes occur. There is an impairment of multiple functions including the dermal, mucosal, and epithelial barrier and (50) the barrier effect (70). While most scientists have not found a quantitative variation in immunological cells with aging, B lymphocytes and T lymphocytes associated with adaptive immunity and natural killers cells, granulocytes, monocytes, and macrophages associated with the innate immunity were found in increased numbers (71, 72).

Yet, in elderly people, DCs showed a reduction in antigen presentation-function, impaired endocytosis, and reduced chemokine production (72). This reduced chemokine production leads to a decrease in cytotoxicity of the natural killer (NK) cells and a decreased killing capacity. While natural killer numbers do increase in healthy elderly people due to the enhanced activity of the markers cells CD56dim and CD57, function is impaired due to cytotoxicity. Therefore, an enhanced production of IL-4 and IL-10 and a decreased production of INF-γ in elderly subjects is observed.

The importance of natural killer (NK) cells in kidney infection and inflammation was previously discussed. Although, natural killer (NK) cells increase quantitatively in lymphatic organs, they showed a low proliferative capacity in the peripheral blood (73). Neutrophils make up 50–70% of human white blood cells and they play an essential role in the innate immune system. They remain stable in the peripheral blood and the bone marrow of the elderly, although they have low phagocytic and killing activity and are more vulnerable to apoptosis (74). Although monocytes also have stable quantitative levels in the peripheral blood of an aged subject, macrophage function is decreased (75, 76). Yet, a temperate phagocytosis, chemiotaxis, and oxidative activity is seen due to the release of ROS, as superoxide radical and hydrogen peroxide from different cells (76). Moreover, the antigen-presenting capacity is lower. Thus, infection occurring in the elderly will be long lasting and it is likely to develop into a chronic inflammation state more frequently. Similarly, the same profile was shown for dendritic cells (DCs) (77). Moreover, in the frail elderly, an extensive reactivity against auto-antigens and an enhanced release of the pro-inflammatory cytokines TNF-a and IL-6 was registered (78). It is of note that these pro-inflammatory cytokines are used as predictive biomarkers for comorbidities and mortality (17).

Shifts observed in the immunological structure during chronological aging induce a “prolific milieu” for the development of a chronic inflammation state, so-called “*inflammaging.”*

Age-related modifications are more pronounced in the adaptive immune system.

Chronological aging lends itself to the decrease of naïve T cells and the accumulation of oligoclonal memory and cytotoxic T cells (79). Upon the end of the thymus involution process at around 50 years of age, a drop in T cell levels is marked and globally observed age-related shifts are more noticeable (80). The decrease of CD8+ cells was more profound compared to CD4+ cell levels (81).

Although, B lymphocytes present a stable profile in the peripheral blood, the numbers of mnemonic B cells is enhanced in order to offset the drop in naïve B cells in the elderly. In support of that observation, insufficient production of specific antibodies following vaccination with advanced aged was shown (82).

Recapitalizing, important shifts are shown in immune system cells during aging which lead to thymic involution, clonal exhaustion, and rupture (83) (Figure 1).

The term immunosenescence was coined by Roy Walford (84) when he published his hallmark book entitled “The Immunologic Theory of Aging” (85). The term denotes the aging-related dysfunction of the immune system (72) associated with higher infection possibility.

However, there is some scientific disputation in defining the term “immunosenescence” (72). Scientists report immunosenescence as a dysfunction of the global immune system called the “damage theory of aging,” while others believe that only specific parameters are altered (72) entangling the telomere proliferation mechanisms (86). Telomeres seem to have a crucial role in aging via regulating cellular responses and DNA damage (87). Telomeres should “cap” chromosome ends to inhibit activation of DNA repair. As a result, apoptosis or cell senescence occurs when the number of “uncapped” telomeres accrues (87) due to shortening of each telomere length. This fact highlights the cessation of cellular proliferation which defines the aging status. Finally, a lack of telomeres is reported as an immunosenescence status (87). It is of note that amplified cancer cells have active telomerases and a stable telomere length and as a result they do not senescence and even when telomeres are linked to oncogenes, cells tend to immortalize (86–88).

Aging is linked to important shifts in gene expression. Overexpression of p16 and p21 gene inhibitors of the cellular cycle induce faster senescence (89). In this way, the induction of senescence induced by gene inhibitors may be a new therapeutic approach in the treatment of cancer (89).

## Chronic Kidney Disease and Immunosenescence

The term “chronic kidney disease (CKD)” reflects lasting damage to the kidneys that can aggravate over time. Chronic kidney disease (CKD) and end-stage renal disease (ESRD) are a dominant medical challenge in the 21st century (90), as more than 1.2 million people died from CKD in 2017 showing a considerable increase in global-age prevalence and mortality in the last 20 years (91). In Oceania, sub-Saharan Africa, and Latin America, the burden of CKD was much higher compared to the disease burden in other countries (91). Patients may develop complications such as hypertension, anemia, heart and blood vessel diseases, and nerve damage (90). Diabetes and hypertension may cause CKD, susceptibility to infection, and other associated pathologies (90).

Early detection of the disease is important, as the disease develops and may lead to kidney impairment that necessities dialysis and finally kidney transplantation (90) to survive.

CKD is defined according to the level of glomerular filtration rate (GFR) into five gradual stages from asymptomatic stage 1 to the end-stage renal disease (ESRD) stage 5. The stages 3–5 show a glomerular filtration rate (GFR) below 60 ml/min per 1.73 m^2^ for 3 months or more (90). Other pathological co-morbidities as well as additional markers of kidney damage, such as proteinuria or hematuria for 3 months or more are co-estimated (92). The disease seems to be more common in the elderly population considering chronological aging. CKD is characterized by senescence, and CKD and ESRD patients appear to be biologically older (93) due to their global malfunction status, when compared to a healthy population.

Kidney cellular shifts and immune cell dysfunction lead to immunosenescence and apoptosis as previously discussed. Moreover, important changes are registered in the kidney glomerular filtration barrier by loss of podocytes (94) which lead to renal impairment. Proteinuria and other lesions advance podocyte loss or induce death (75, 94).

The autophagy process has a central role in controlling homeostasis and adjacent involved mechanisms involved in glomerular disease and maintains podocytes homeostasis in aging (95, 96).

Tubulo-interstitial renal fibrosis is a condition of the aged kidney which is defined as a progressive detrimental connective tissue deposition on the kidney parenchyma leading to renal function damage (43, 97). The epithelial to mesenchymal transition (EMT) of tubular epithelial cells is converted to mesenchymal fibroblasts. Thus, fibroblasts proliferate to the adjacent interstitial parenchyma (97) (Figure 1).

As stated, advanced aging deteriorates the immune system, increases susceptibility to infection (98), and converges a low-grade activation of the inflammation system called inflammaging (79). Stimuli such as exposure to pathogens, cellular debris, nutrients, and the gut microbiota sustain inflammaging (53, 99).

The gut microbiota is the corner stone in inflammaging due to its capacity to produce inflammatory products and dialogue with other organs and systems (54). However, it is clear that the underlying aging mechanisms still need to be explained through this trajectory in order to gain a better understanding of this global dysregulation and provide more effective therapeutic approaches.

## Author Contributions

ES: conceptualization, writing, and editing. KK and CT: formal analysis and writing. CV and KA: resources and writing. TK: design and editing. MCC: writing and editing. EB and ET: supervision, original draft preparation, and editing. ET contribute to the reviewing and editing of the paper. All authors contributed to the article and approved the submitted version.

## Conflict of Interest

The authors declare that the research was conducted in the absence of any commercial or financial relationships that could be construed as a potential conflict of interest.
